# Water-seeking behavior among terrestrial arthropods and mollusks in a cool mesic region: Spatial and temporal patterns

**DOI:** 10.1371/journal.pone.0260070

**Published:** 2021-11-22

**Authors:** Jamie E. Becker, Nadejda A. Mirochnitchenko, Haley Ingram, Ashley Everett, Kevin E. McCluney

**Affiliations:** Department of Biology, Bowling Green State University, Bowling Green, Ohio, United States of America; University of Cincinnati, UNITED STATES

## Abstract

Dehydration can have negative effects on animal physiological performance, growth, reproduction, and survival, and most animals seek to minimize these effects by reducing water losses or seeking water sources. Much—but not all—of the research on animal water balance comes from dryland ecosystems. However, animals inhabiting mesic regions may also experience desiccating conditions, for example within urban heat islands or during heatwaves and droughts. Here we examined how spatial variation in impervious surface and spatial and temporal variation in microclimate impact water demand behavior of terrestrial arthropods and mollusks in three areas of mesic Northwest Ohio, with analysis of taxa that exhibited the greatest water demand behavior. Water demand behavior was measured as the frequency that individuals were observed at an artificial water source (a moistened pouch), relative to the frequency at a control (a dry pouch). Overall, terrestrial arthropods and mollusks were found about twice as often at the water source than at the control (equivalent to 86 more observations on the wet pouch than on dry at each site, on average), with ants accounting for over 50% of the overall response in urban areas. Daily fluctuations in vapor pressure deficit (VPD) best predicted daily variation in water demand behavior, with increased demand at higher VPD. Mean VPD was generally highest near urbanized areas, but effects of VPD on water demand behavior were generally lower in urbanized areas (possibly related to reductions in overall abundance reducing the potential response). On certain days, VPD was high in natural areas and greenspaces, and this coincided with the highest arthropod water demand behavior observed. Our results suggest that terrestrial arthropod communities do experience periods of water demand within mesic regions, including in greenspaces outside cities, where they appear to respond strongly to short periods of dry conditions—an observation with potential relevance for understanding the effects of climate change.

## Introduction

Insects and other terrestrial arthropods are susceptible to water loss because of their small body size, and hot, dry conditions exacerbate this problem [1, 2]. For instance, evaporative water loss increases when the concentration of water vapor in the air decreases, and respiratory water losses also increase, for ectotherms, as metabolic rates increase at higher ambient temperatures [1, 3]. Terrestrial arthropods exhibit various evolutionary and physiological mechanisms that combat dehydration [4–11]. Behavioral plasticity is another important method by which arthropods may persist in desiccating environments. For example, arthropods may maintain water balance by becoming less active [12], by decreasing exposure to high temperatures [13, 14], by altering nutrient consumption to favor metabolic water production [15], and by regulating intake of free water and moist food [16–19].

Dehydration and altered behavioral patterns resulting from increased water demand can have important ecological consequences, and this topic is relatively well-studied in arthropods inhabiting xeric regions. For instance, dehydration can decrease muscle performance [13], growth rate [5, 20], survival [21, 22], and reproduction [23, 24]. Behavioral adaptations to combat increased water demand can ultimately affect species distributions [25], species richness [26, 27], community composition [6, 28, 29], and trophic interactions [17, 30–33]. However, the frequency of water demand and its ecological consequences in mesic regions has been less investigated [15, 19, 34].

Mesic regions may have specific locations and time periods that lead to increased animal water demand. First, animals living in sandy areas may have increased water demand because water readily percolates through wider pores in sandy soils, lowering the soil moisture at the surface [35]. Second, animals living in urban areas may show increased water demand because cities can reduce the abundance of water bodies [36–38] or increase temperatures locally (urban heat islands), which can increase the rate of evaporation, reducing soil moisture and potentially directly increasing animal water loss rates [39–41]. Finally, animals may experience increased water demand in most mesic environments during certain periods of time, like heatwaves and droughts [42, 43]. These climatic events may have a greater effect on terrestrial arthropods inhabiting mesic environments that are not well-adapted to desiccating conditions (hypothesized by McCluney [33]).

In this paper, we investigated the frequency of water-seeking behavior among terrestrial arthropods and mollusks in mesic Northwest Ohio, by making repeated observations of their presence at an artificial water source (a moistened pouch), relative to a control (a dry pouch). We predicted that this water-seeking behavior would occur more frequently within landscapes with high amounts of impervious surface, because these areas tend to create hot and/or dry microclimates that promote desiccation [40, 41]. Second, we examined how strongly temporal variation in water demand behavior is linked to environmental factors (e.g., temperature, vapor pressure deficit, and soil moisture), including both spatial patterns in average climatic differences, which covary with landscape conditions, and temporal patterns in weather conditions, which vary daily. Finally, we examined taxonomic variation in this type of water-seeking behavior, because different taxa of arthropods and mollusks likely have different physiological and behavioral adaptations to limit desiccation.

## Materials and methods

We selected three areas in Northwest Ohio differing in size and impervious surface: Toledo (a medium-sized city, 40–80% impervious surface within sites), Bowling Green State University (BGSU; college campus in a small town, 30–90% impervious surface within sites), and Oak Openings (nature preserve, 0% impervious surface within sites; S1 Fig). In Toledo and BGSU, we contrasted street trees to trees in nearby greenspaces. Within Oak Openings, we contrasted trees in a moderately well-drained sandy soil to those in a very poorly drained soil with a higher clay content.

We placed two pairs of wet and dry water pillows (small pouches filled with a polymer that absorbs water; Cricket water pillows, Zilla, Franklin, WI; used in previous studies [15, 17, 19, 32]) at ten trees at each location at 3PM–one pair in the branches, and one pair at the tree’s roots. Wet water pillows were hydrated with deionized water (containing ~30 mL) and placed within two inches of (but not touching) the paired dry pillow, with the water accessible side up, attached to a binder clip to prevent pillows from blowing away (or to attach to a branch). From 5 June to 8 August 2014, we visited each area once every three days (13 visits), during which we recorded and photographed the arthropods detected on the water pillows in the late afternoon (4PM) and at night (10PM) on the same day. New pillows were placed at the start of each observation day and collected at the end of each observation period and thus pillows were left out for a total of 7–8 hours each day and did not dry out during this period. Pillows were not placed or visited during storm events, and it did not storm unexpectedly during this study and thus dry pillows did not become unexpectedly moist.

Using a hand-held weather station (WS-HT350, Ambient Weather, Chandler, AZ), we measured shaded temperature and relative humidity once per visit. We measured volumetric soil moisture three times per tree (SM 150 soil moisture sensor, Dynamax, Houston, TX) at the highest, lowest, and medium points of uneven soil within 0.5m of the tree. Because the arthropods and mollusks examined in this study could likely travel to patches of moist soil within this area, we used the maximum soil moisture per tree as a metric of ambient water availability in further analyses. We calculated vapor pressure deficit (VPD) from temperature and relative humidity using the ASCE standardized reference evapotranspiration equation [44]. We also calculated percent impervious surface per tree within a 25m radius using the National Land Cover Database 2011 [45].

Because abundance data could have inflated terrestrial arthropod visitation at each tree (especially in the case of colonial ants), we calculated water demand as the frequency of observing at least one individual. First, we calculated a frequency of water demand per tree over our sampling period using the number of observation dates with at least one terrestrial arthropod or mollusk present, out of 13 visits per tree (hereafter “frequency per tree”), allowing us to examine patterns related to differences in impervious surface. Second, we calculated the frequency of water demand per day, across all trees at a site, using the number of trees with at least one terrestrial arthropod present, out of 10 trees per site (hereafter “frequency per day”), allowing us to examine the effects of daily weather variation. Frequency per tree and frequency per day included any arthropod or mollusk, but we also calculated the frequency of observations of arthropods within specific taxonomic groups. Terrestrial snails and slugs (i.e., Panpulmonata) were included in our calculations when they were observed. Observations from the ground and from branches, as well as observations from the afternoon and evening were combined to reduce complexity of analysis and difficulty with interpretation (S2 Fig).

To examine the effect of impervious surface, we used binomial generalized linear mixed effects models, with observation frequency per tree as the response and water pillow type (wet, dry) and percent impervious surface as fixed factors, and area (Toledo, BGSU, Oak Openings), site (street vs. greenspace or sandy vs. clay), and tree as nested random factors. To test the effect of urbanization (a single variable of interest) on animal water demand, we compared the full model containing impervious surface to a null model without impervious surface (but with the other “nuisance” variables), using a parametric bootstrapping method to derive p-values, following [46, 47]. Bootstrapping is a statistical procedure that resamples a single dataset to create many simulated samples, and p-values obtained from this method are reported to being the most reliable [47]. Our comparison tested 1000 simulations and used the *lme4*, *arm*, and *pbkrtest* packages. We did not explicitly examine how spatial variation in microclimate (temperature, VPD) influenced spatial variation in water demand behavior, because we only had one measurement location of temperature and VPD per site, preventing our ability to make inferences about these spatial patterns.

We used AIC and binomial generalized mixed models (see above) to compare the relative importance of both spatial variation in mean environmental conditions (climate, including temperature, VPD, and soil moisture) and temporal variation (weather, including temperature, VPD, and soil moisture) in predicting water demand behavior. To examine the influence of spatial patterns of microclimate on temporal variation in water demand behavior, we calculated averages of all temporal measurements of temperature, VPD, and soil moisture (referred to as “mean site temperature,” “mean site VPD,” and “mean site soil moisture”). To examine temporal patterns of microclimate we calculated averages of all spatial measurements within each site (across the 10 trees), per day, for the same measured environmental variables (referred to as “daily temperature,” “daily VPD” and “daily soil moisture”).

To examine potential predictors of changes in water demand behavior over time (daily), we first used normalized linear mixed models to compare alternative temporal autocorrelation structures–those that assumed compound symmetry or autoregressive variance-covariance–and picked the best model, using AIC. Compound symmetry was verified as the better model, allowing subsequent use of binomial generalized linear mixed effects models with observation frequency per day as the response, interactive comparisons between a single environmental variable and pillow wetness as fixed factors, and area and site as nested random factors. For each response metric, we considered models within 2 AIC units to be equivalent. Candidate models were analyzed within the *lme4* and *AICcmodavg* packages. Examinations of temporal autocorrelation used linear mixed effects models in the *nlme* package, since this package does not allow for generalized models.

For linear mixed models, assumptions of normality and equality of variance were checked using normal probability plots on residuals and graphs of residuals vs. fitted estimates, respectively. Multicollinearity of environmental variables was assessed by observing variance inflation factors, none of which were greater than 5. Analyses were conducted in R v. 4.0.2.

### Ethics statement

Scientific collection of arthropods was authorized under Ohio Division of Natural Resources permit number 17–204. Toledo Parks and Recreation, The Nature Conservancy, and Bowling Green State University provided permission to conduct experiments on their land. No animals were collected or harmed during this project, and no endangered or protected species were at risk.

## Results

Overall, terrestrial arthropods and mollusks were present on wet pillows for a median of 23.3% of observations across locations, versus 12.3% on dry pillows, a significant difference of 11 percentage points (χ^2^ = 36.0, *P* < 0.01), which translates to 86 more observations on wet pillows than on dry pillows. Additionally, the difference in occurrence on wet and dry pillows was, on average, larger within the clay site and within greenspaces (Table 1). Differences in occurrence between wet and dry pillows varied from 0 to 53.8 percentage points at each individual tree.

**Table 1:** Results comparing water demand behavior among sites.

Region	Site	Dry pillow ^a^	Wet pillow ^a^
Oak Openings	Sand	33.08 ± 5.00%	43.85 ±4.45%
	Clay	13.99 ± 3.89%	37.69 ± 4.21%
BGSU	Street trees	3.85 ± 1.72%	7.69 ± 2.81%
	Greenspace	11.11 ± 3.24%	25.38 ± 5.85%
Toledo	Street trees	3.85 ± 1.72%	6.15 ± 1.54%
	Greenspace	7.69 ± 1.62%	19.23 ± 4.17%

^a^Values show mean and standard error of observation frequency per tree.

**Fig 1:**
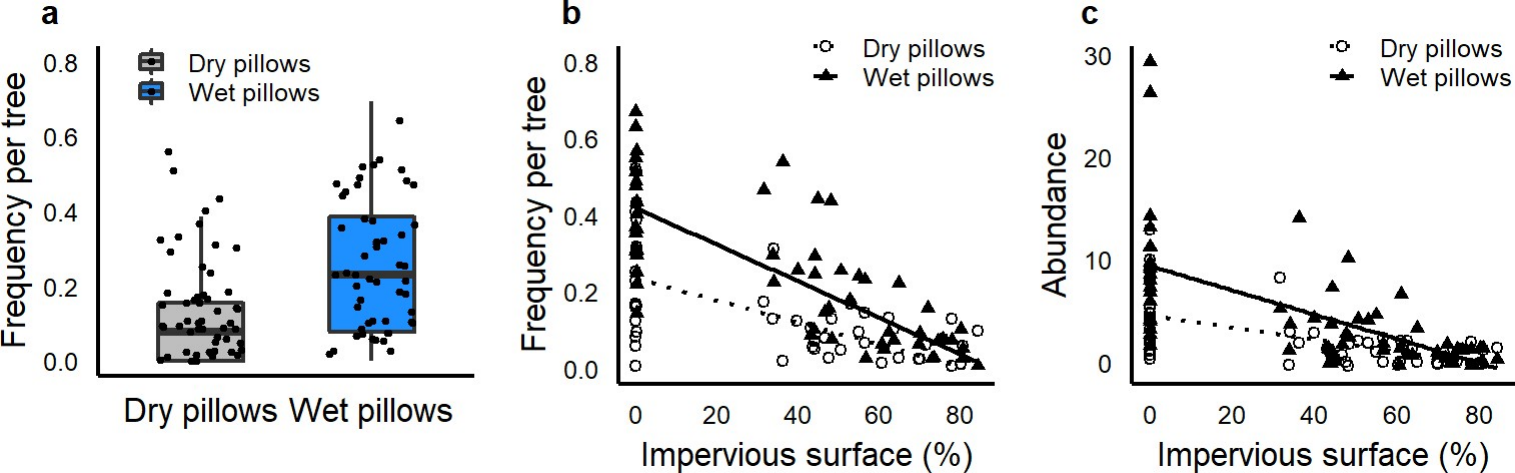
Relationship between spatial variation in arthropod and mollusk occurrence on water pillows of different wetness and impervious surface. Impervious surface is calculated within a 25m radius circle around each tree where water pillows were located. (a) Terrestrial arthropods and mollusks were observed on wet pillows about twice as often as on dry pillows (23.3% vs 12.3% of observations). The frequency of observations (b) and the total abundance (c) of arthropods on both wet and dry pillows declines with increased impervious surface.

The frequency of observations of animals per tree (representing spatial variation) declined with increased impervious surface, (χ^2^ = 28.6, *P* < 0.01; *R*^2^ = 0.48; Fig 1B), with frequency on wet pillows much higher than dry at low impervious sites, but frequency on wet and dry pillows converging on a low value at high impervious sites (i.e., few observations on any pillow type with high impervious surface).

**Fig 2:**
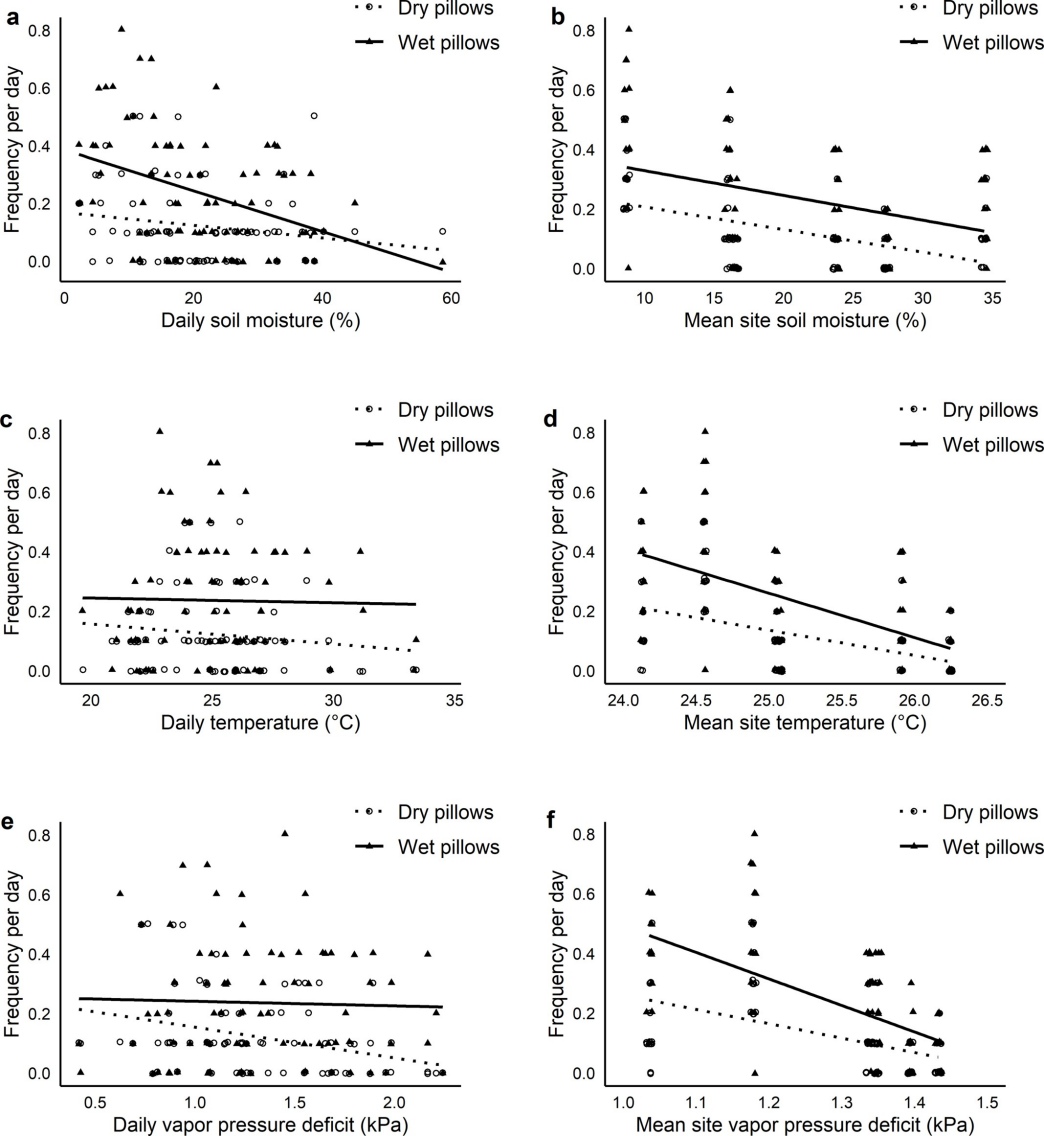
Associations between each measured environmental variable and the temporal variation in frequency of observations of terrestrial arthropods and mollusks on wet and dry water pillows. Each figure depicts regression lines of best fit from a linear model relating each environmental variable to the frequency of observation of terrestrial arthropods and mollusks for the wet and dry pillow separately. They are useful in visualizing the patterns in the data. The most parsimonious model was one that included interactive effects of mean site temperature (d), daily vapor pressure deficit (e), and mean site daily vapor pressure deficit (f).

The strongest environmental predictor (the most parsimonious model) of the temporal variation in differences in observations of terrestrial arthropods and mollusks on wet and dry pillows was daily VPD, where an increase in daily VPD led to increased water demand, as defined by a greater difference between wet and dry pillow observations (Fig 2 and Table 2A); but this model had relatively low explanatory power (marginal *R*^2^ = 0.07). When using the explanatory term from the first set of candidate models to explore potential interactive and additive effects of various environmental predictors in a second multi-model comparison (Table 2B), two models were equally parsimonious, where increases in daily VPD increase water demand more frequently as mean site temperature and mean site VPD decrease. These models had higher explanatory power (marginal *R*^2^ = 0.19 and 0.21, respectively) than the influence of daily VPD alone.

**Table 2:** Results comparing a set of candidate models to find the best predictor for variation in water demand behavior over time.

Model	K^a^	AICc^b^	ΔAICc^c^	Weight^d^	LL^e^	*R* ^2^ ^f^
(a) Frequency per day						
~ Pillow ˟ Daily VPD^g^	6	469.82	0.00	0.79	-228.63	0.07
~ Pillow ˟ MSVPD^h^	6	474.20	4.38	0.09	-230.82	0.17
~ Pillow	4	476.11	6.29	0.03	-233.92	0.05
~ Pillow ˟ MST^i^	6	476.33	6.51	0.03	-231.88	0.15
~ Pillow ˟ Daily temperature	6	477.23	7.41	0.02	-232.33	0.05
~ Pillow ˟ Daily soil moisture	6	477.24	7.42	0.02	-232.34	0.05
~ Pillow ˟ MSSM^j^	6	478.13	8.31	0.01	-232.78	0.09
~ Null	3	512.60	42.78	0.00	-253.22	0.00
(b) Frequency per day						
~ Pillow ˟ Daily VPD ˟ MST	10	466.62	0.00	0.46	-222.55	0.19
~ Pillow ˟ Daily VPD ˟ MSVPD	10	466.82	0.21	0.42	-222.65	0.21
~ Pillow ˟ Daily VPD	6	469.82	3.20	0.09	-228.63	0.07
~ Pillow + Daily VPD + MSVPD	6	473.12	6.50	0.02	-230.28	0.17
~ Pillow + Daily VPD + MST	6	475.27	8.66	0.01	-231.35	0.15
~ Pillow ˟ Daily VPD ˟ MSSM	10	476.91	10.30	0.00	-227.70	0.11
~ Pillow + Daily VPD	5	477.27	10.65	0.00	-233.44	0.05
~ Pillow + Daily VPD + MSSM	6	478.60	11.98	0.00	-233.02	0.09
~ Null	3	512.60	45.99	0.00	-253.22	0.00

^a^Number of model parameters.

^b^Akaike information criterion (AIC). The lower-case ’c’ indicates that the value has been corrected for small sample sizes.

^c^The relative difference between the most parsimonious model (which has a ΔAIC of zero) and each other model in the set. For each response metric, we considered models within 2 AIC units to be equivalent.

^d^Akaike weight, which gives the probability that the model is the best from the set.

^e^Log-likelihood value.

^f^Marginal *R*^2^ value.

^g^Vapor pressure deficit (kPa).

^h^Mean site vapor pressure deficit (kPa).

^i^Mean site temperature (˚C).

^j^Mean site soil moisture (% vol).

**Fig 3:**
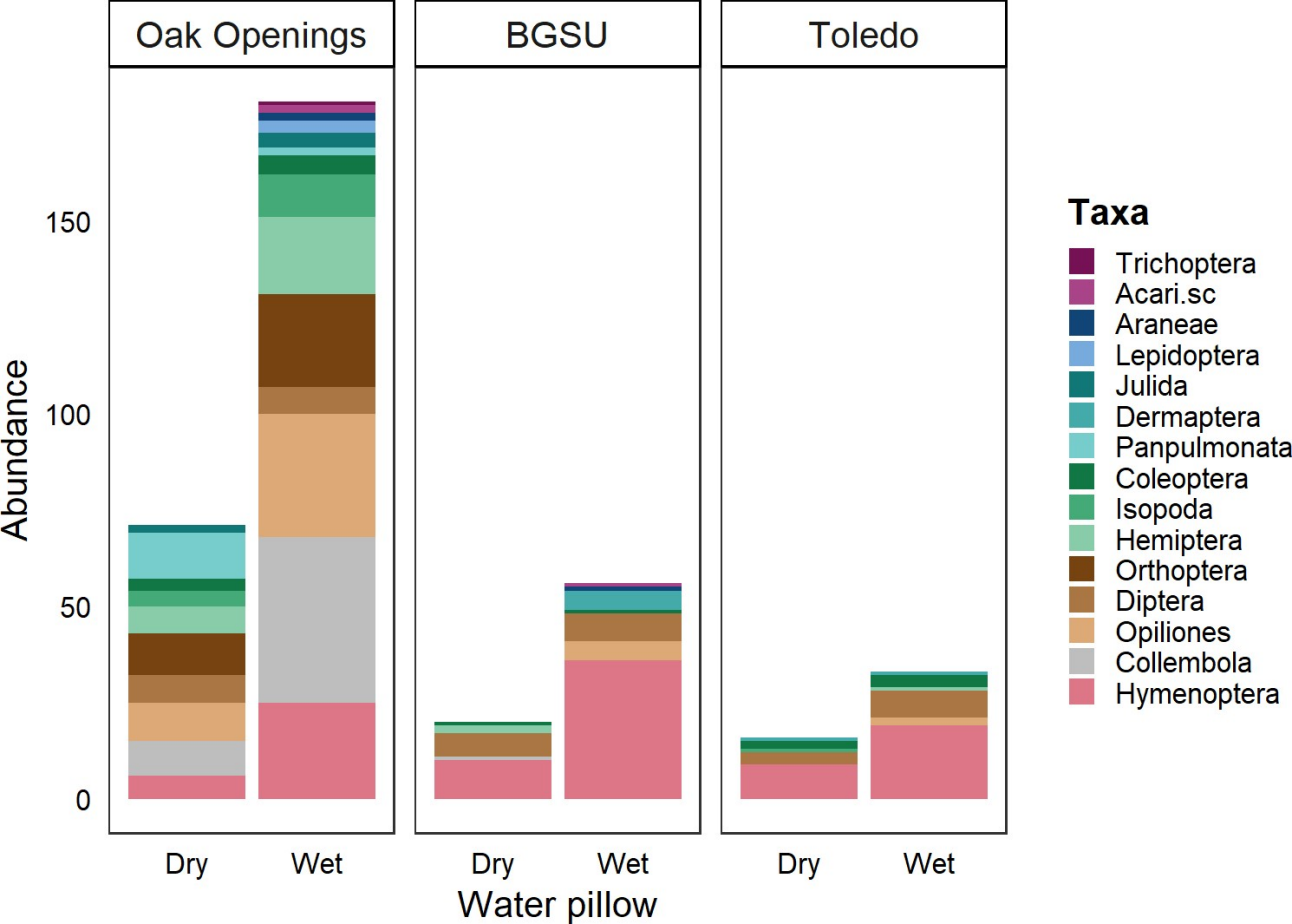
Total abundance of terrestrial arthropods and mollusks on wet and dry pillows. Responses to water pillows were dominated by Opiliones, Orthoptera, Hymenoptera, and Collembola at the undeveloped sites (Oak Openings), while developed sites (BGSU and Toledo) were primarily composed of Hymenoptera (mostly ants, especially *Camponotus*, *Lasius*, and *Brachymyrmex*). These genera are widespread in OH and were present at all sites. Ants in Toledo and BGSU were primarily *Camponotus pennsylvanicus*.

The overall response at the nature preserve (Oak Openings) was primarily dominated by Opiliones, Orthoptera, Hymenoptera, Hemiptera, and Collembola. Of the 15 Orders observed in this study, Oak Openings (nature preserve) contained 93% of the taxa (lacking Dermaptera), with 50% at BGSU (college campus in small town) and 43% in Toledo (downtown in a medium-sized city). Across all areas, ants accounted for 38% of the total response on wet and dry pillows. At BGSU and in Toledo, ants accounted for 62.76% and 57.4% of the total response, respectively (Fig 3), and they were found in similar abundances on wet and dry pillows in these more urban locations.

## Discussion

Overall, our results suggest that terrestrial arthropods and mollusks in mesic regions do experience periods of increased water demand behavior despite the relatively cool and moist average conditions. Water demand behavior increases when the air is more desiccating (daily VPD increases), especially in greenspaces and soils with higher clay content (Tables 1 and 2), and for particular taxa, like ants (Fig 3).

We expected terrestrial arthropods living within cities in mesic regions to be more water-limited than those living in natural areas, because the urban heat island effect increases ambient temperatures and increases VPD [39–41]. Such changes in temperature and VPD are known to increase cuticular water loss [1, 2], furthering our expectations of increased water limitation with increased impervious surface. However, we did not find support for these hypotheses. Instead, we found that water demand behavior was more commonly observed in undeveloped areas and less commonly observed with increasing impervious surface (Fig 1B). But this result may have been driven by a corresponding decline in abundance with increasing impervious surface (Fig 1C). Additionally, our ability to make strong conclusions about the effects of impervious surface are hampered by the limited number of locations studied. However, these results suggest that taxa in generally cool, moist sites can be responsive to daily increases in VPD, increasing their water demand behavior.

Although our results have multiple possible explanations, one possibility is that higher water demand behavior at cool and moist sites could be partly due to taxa at those sites having fewer adaptations to xeric conditions (hypothesized by McCluney [33]). For example, Yilmaz et al. [48] found that rearing temperature positively influenced urban and rural isopod body size, which consequently improved desiccation tolerance via reduced cuticular water loss. Kaiser et al. [49] also showed that urbanized sites tended to produce larger male *Lasiommata* butterflies (a thermophilic species), although no such plasticity occurred in the woodland butterfly. Apart from body size, another organismal trait commonly seen in mesic-adapted arthropods is that they have thinner, more permeable cuticles with fewer hydrocarbons [1, 50, 51]. Evolutionary adaptations to environmental conditions can cause more xeric-adapted “water conserver” species to be present in cities and more mesic-adapted “water seeker” species to be present in undeveloped locations [33, 48, 49, 52–55]. Thus, although terrestrial arthropods in mesic regions (and cooler sites) may infrequently experience water limitation, they may be more greatly affected when droughts or heatwaves occur (hypothesized in McCluney [33]). Our results support this hypothesis, as indicated by the interactive effects of increased daily VPD, but decreased mean site temperature and decreased mean VPD, on water demand behavior (although the *R*^2^ value was not strong). However, greater examination of differences in community composition and functional traits is needed to test this idea.

Although we did not attempt to explicitly link short-term changes in water demand behavior to either physiological condition or abundance, others have found that periods of increased water demand behavior can have food web consequences. For example, in other research we found that changes in water balance associated with urbanization can influence arthropod demand for particular macronutrients [15]. Additionally, McCluney and Sabo [32] demonstrated that water demand can alter both direct and indirect species interactions, influencing trophic cascades. Moreover, we documented a strong response among ants (Fig 3), which have been shown to play important roles in food webs and ecosystems in and outside of cities, including roles in altering waste removal and pest abundance [56–58]. Therefore, water demand could alter food webs in mesic regions in ways that have important consequences for people.

We note that our research does have several caveats. First, water pillows might provide localized cooling in addition to a water source. Previous research has observed changes in water content of arthropods with the presence of wet water pillows (McCluney et al 2018), but we did not explicitly examine that here. Second, it is possible that predators may have been attracted to prey on the water pillows. However, our response metric was the frequency of observing at least one arthropod or mollusk on a pillow and thus this metric should not be influenced by predators being attracted to prey present on the pillows. Third, we made our observations at a limited number of developed locations. To better understand how urbanization might influence water demand would require examination of patterns across a greater number of sites that vary in impervious surface.

Overall, this study suggests that terrestrial arthropods inhabiting a cool, mesic region exhibit water demand behavior, and this behavior seems to increase with daily vapor pressure deficit and, counterintuitively, does so more strongly at cool, humid sites and those with less impervious surface. It is possible that droughts may more strongly affect food webs in cooler, more mesic locations than in more xeric ones, even if desiccating conditions are experienced less frequently, because of greater abundances of organisms and fewer adaptations to reduce desiccation. Climate change projections indicate increased intensity of droughts and heatwaves worldwide [42, 43], and our work suggests these events might have disproportionate ecological effects in cooler parts of mesic regions. Future work is needed to more closely link specific organismal traits and environmental conditions with water demand behavior.

## Supporting information

S1 Fig Nested map of each area. In Toledo and BGSU, we contrasted street trees [Toledo (41°39’17.3"N, 83°32’04.9"W), BGSU (41°22’54.0"N, 83°38’28.8"W)] to trees in greenspaces. [Toledo (41°39’23.4"N, 83°32’09.0"W), Bowling Green (41°22’49.9"N, 83°38’29.3"W)]. Within Oak Openings, we selected trees at a site which had sandy soil (41°37’45.91"N, 83°47’5.45"W), and at a site which had clay soil (41°37’44.09"N, 83°48’45.89"W). This figure was created by the authors using Web Soil Survey [59] for illustrative purposes only. No copyrighted material was used.
(PNG)

S2 Fig Frequency of observations with pillow location and time of day. Arthropods were observed significantly more often at night than during the evening (χ^2^ = 33.8, *P* < 0.01), but the frequency of observations on wet pillows, compared to dry pillows, was also significant (χ^2^ = 49.0, *P* < 0.01) with no interactive effects. Arthropods were also observed significantly more often on the ground than in tree branches, but this interacted significantly with pillow wetness (χ^2^ = 9.8, *P* < 0.01). Finally, flying insects were often observed on the ground while ants were often observed in tree branches. Thus, we combined these data to reduce the complexity of our models and to improve interpretation.
(PNG)
