# Supplementary figures and images for: Water-seeking behavior among terrestrial arthropods and mollusks in a cool mesic region: Spatial and temporal patterns
Source: PLoS One. 2021 Nov 22;16(11):e0260070. doi: 10.1371/journal.pone.0260070 (PMC8608307; doi:10.1371/journal.pone.0260070)

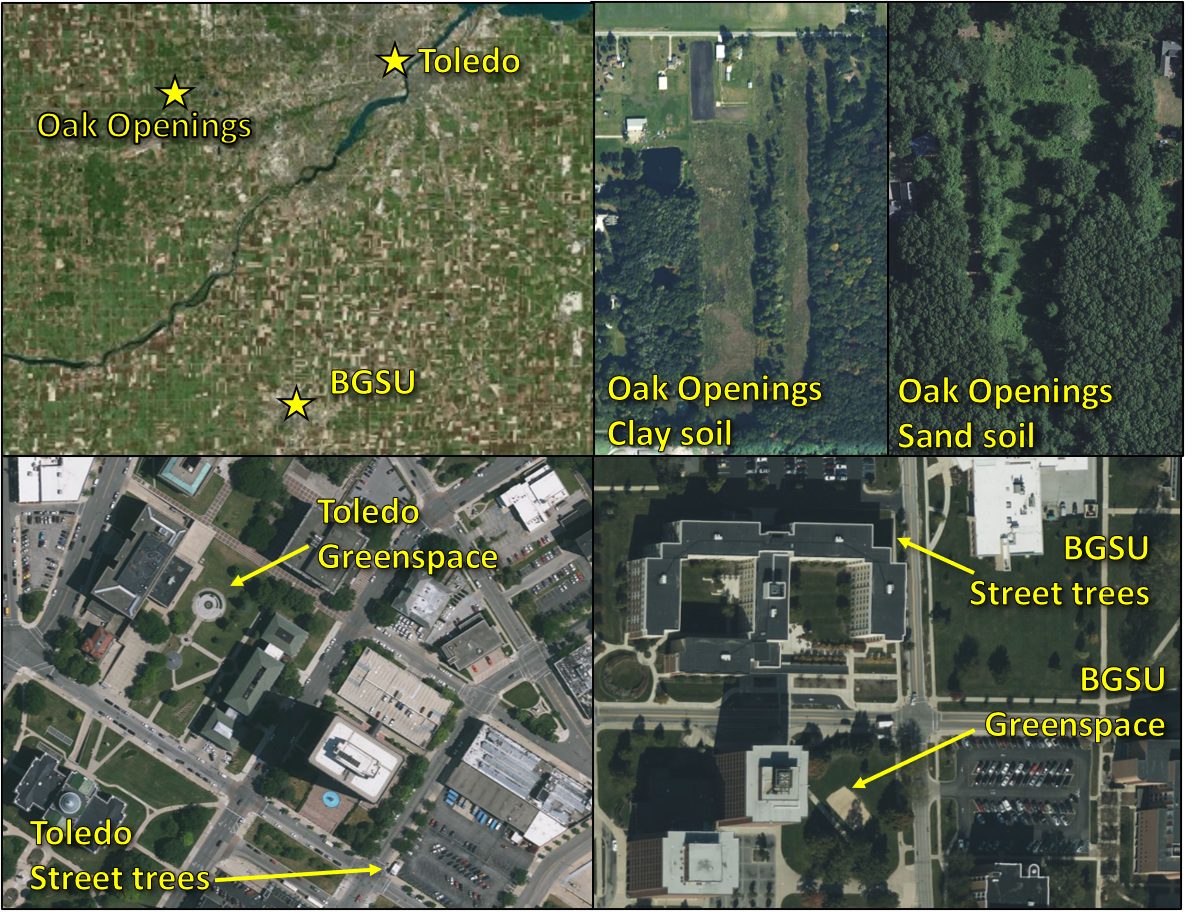

Supplement: S1 Fig — In Toledo and BGSU, we contrasted street trees [Toledo (41°39’17.3"N, 83°32’04.9"W), BGSU (41°22’54.0"N, 83°38’28.8"W)] to trees in greenspaces. [Toledo (41°39’23.4"N, 83°32’09.0"W), Bowling Green (41°22’49.9"N, 83°38’29.3"W)]. Within Oak Openings, we selected trees at a site which had sandy soil (41°37’45.91"N, 83°47’5.45"W), and at a site which had clay soil (41°37’44.09"N, 83°48’45.89"W). This figure was created by the authors using Web Soil Survey [59] for illustrative purposes only. No copyrighted material was used. (PNG) [file pone.0260070.s001.png]

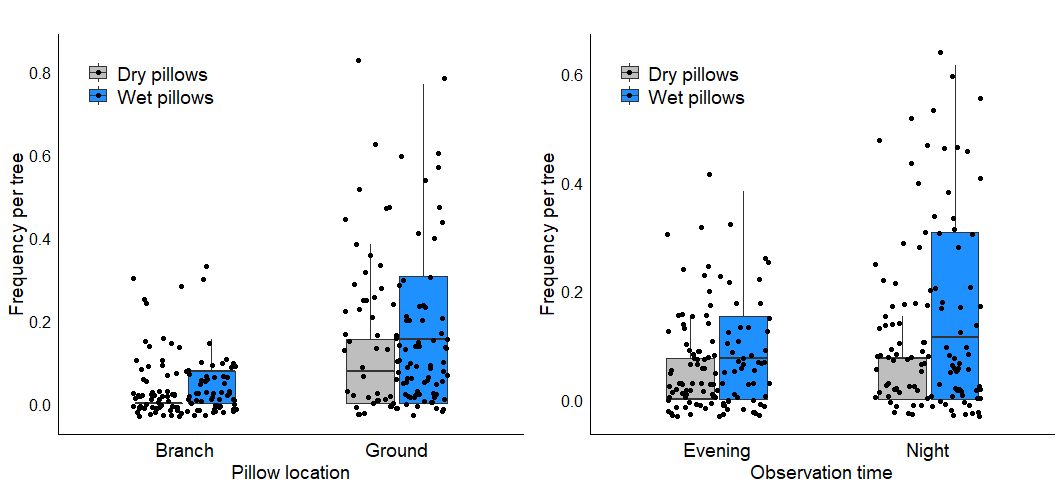

Supplement: S2 Fig — Arthropods were observed significantly more often at night than during the evening (χ2 = 33.8, P < 0.01), but the frequency of observations on wet pillows, compared to dry pillows, was also significant (χ2 = 49.0, P < 0.01) with no interactive effects. Arthropods were also observed significantly more often on the ground than in tree branches, but this interacted significantly with pillow wetness (χ2 = 9.8, P < 0.01). Finally, flying insects were often observed on the ground while ants were often observed in tree branches. Thus, we combined these data to reduce the complexity of our models and to improve interpretation. (PNG) [file pone.0260070.s002.png]
